# Analysis of diagnostic delay and its influencing factors in patients with chronic obstructive pulmonary disease: a cross-sectional study

**DOI:** 10.1038/s41598-021-93499-9

**Published:** 2021-07-09

**Authors:** Zhongshang Dai, Yiming Ma, Zijie Zhan, Ping Chen, Yan Chen

**Affiliations:** Department of Respiratory Medicine, Second Xiangya Hospital, Central South University, Changsha, China

**Keywords:** Diseases, Medical research

## Abstract

To explore the status of diagnostic delay and to clarify its potentially influencing factors in patients with chronic obstructive pulmonary disease (COPD). A cross-sectional study was conducted in a Chinese tertiary hospital between July 2019 and February 2020. A total of 408 eligible outpatients with COPD were recruited, and relevant data were collected in the form of questionnaires. Diagnostic delay was compared among different characteristics using the Wilcoxon test and Kruskal–Wallis H test. Multivariable linear regression analysis was performed to determine the factors related to diagnostic delay. The median (interquartile range [IQR]) duration of diagnostic delay was 230 (50–720) days. The proportions of COPD patients who chose tertiary, secondary, and first-level hospitals for the first visit were 53.7%, 29.9%, and 16.4%, respectively. Additionally, the proportions of patients who underwent pulmonary function tests for the first visit in tertiary, secondary, and first-level hospitals were 74.0%, 24.6%, and 1.5% (*p* < 0.001), respectively. In terms of characteristics related to diagnostic delay, there was a significant difference in residence, resident manner, COPD assessment test (CAT) score, modified Medical British Research Council (mMRC) dyspnea scale, age, forced expiratory volume in one second (FEV1) % predicted, and years of education (all *p* < 0.01). Linear regression analysis showed that significant predictors of diagnostic delay included FEV1% predicted (*p* < 0.05), resident manner (*p* < 0.001), and years of education (*p* < 0.01). Our study indicates that varying degrees of diagnostic delay may exist in patients with COPD. Measures are needed to intervene in the potential factors associated with diagnostic delay.

## Introduction

Chronic obstructive pulmonary disease (COPD) has become a global public health challenge due to its high prevalence and related mortality^1^. According to the 2013 Global Burden of Disease (GBD 2013) report, COPD has become the third leading cause of death worldwide, with approximately 3 million deaths each year^2^. In China, the results of a latest national survey in 2018 have shown that there are close to 100 million COPD patients, and the prevalence rate in people over 40 years old is 13.7%^3^. Consequently, early diagnosis and intervention of COPD are of great value for preventing the progression of the disease^4^. Several studies have demonstrated that early intervention in patients with COPD may improve lung function^5–7^; the UPLIFT study showed that early intervention could reduce the number of acute exacerbations of COPD^5^. However, many factors such as physician-related factors, patient-related factors, and the heterogeneity of the disease itself may have an impact on the early diagnosis of COPD^8^. COPD patients generally have insufficient knowledge of the disease and a low awareness rate^9^, which may work together with other factors to cause delays in the diagnosis and intervention of COPD. The China Pulmonary Health (CPH) study showed that only 2.6% of patients diagnosed with COPD by pulmonary function test knew they had COPD^3^. A study from South Korea indicated that among male smokers, only 23.8% were aware of COPD, while 30.5% had undergone lung function tests^10^. Another study from Denmark showed that 28% of the smokers did not consider COPD to be a fatal disease^11^.

Medical seeking behaviour refers to the whole process from perceiving symptoms to seeking medical care for the purpose of prevention or early detection of a disease and its treatment^12^. Proper medical seeking behaviour is an important measure for managing an illness and its early treatment, and it also plays a key role in disease transmission and disease control. Diagnostic delay refers to the time interval between the onset of symptoms and the date of diagnosis^13^, and it is one of the most important indicators for evaluating medical seeking behaviour. Wang et al.^14^ found that lower awareness and higher age of tuberculosis patients is associated with delayed visits to medical institutions.

However, there have been no studies on the diagnostic delay and the factors influencing it in patients with COPD in China. Considering the large population of COPD patients in China, we aimed to conduct a comprehensive analysis to investigate the status of diagnostic delay of COPD patients and clarify its potentially influencing factors, which may be of great value in the early diagnosis and intervention of COPD.

## Methods

### Study design

This is a cross-sectional study of outpatients with COPD in a large Chinese tertiary teaching hospital. Ethical approval was obtained from the ethics committee of the Second Xiangya Hospital, Central South University. Informed consent was obtained from all the study participants. All methods were performed in accordance with the ethical standards formulated in the Helsinki Declaration.

### Study participants and procedures

Patients were included if they had been diagnosed with COPD according to the GOLD 2019 criteria^15^, and the age ranged from 35 to 80 years. All patients visited the respiratory outpatient department of the Second Xiangya Hospital from July 2019 to February 2020 and were in a stable condition. The eligible criteria were as follows: (1) who had a confirmed spirometry-verified COPD per the GOLD 2019 report, which was defined as post-bronchodilator forced expiratory volume in one second (FEV1)/ forced vital capacity (FVC) < 0.70; (2) who had no primary diagnosis of other respiratory diseases other than COPD (including asthma, bronchiectasis, pulmonary tuberculosis, pneumonia, pulmonary interstitial fibrosis, lung cancer, pulmonary artery hypertension, and pulmonary embolism, etc.); (3) who had no severe heart, brain, liver, kidney, and hematopoietic diseases other than respiratory diseases; (4) able to complete the questionnaire; (5) no missing data of FEV1, FVC, modified Medical British Research Council (mMRC) scale, COPD Assessment Test (CAT) score, or diagnostic delay.

We performed the study in the form of questionnaires, including a self-made questionnaire, CAT questionnaire, and mMRC dyspnoea scale. Face-to-face interviews were conducted among participants by specially-assigned persons. The self-made questionnaire included sex, age, number of acute exacerbations in the previous year, smoking pack-years, FEV1% predicted, FEV1/FVC, residence, resident manner, years of education, medical insurance, diagnostic delay, hospital classification for first seeking-treatment visit, and previous history of pulmonary function test. Diagnostic delay was defined as the time interval from the onset of symptoms to physician-defined diagnosis of COPD, and it was expressed in days.

### Statistical analysis

Data were analysed using Statistical Package for Social Sciences (SPSS) version 21.0. The data in this study were non-normally distributed after the normality test. Descriptive data without normal distribution were expressed as medians (interquartile range [IQR]), and frequencies were expressed as numbers (percentage). The Wilcoxon and Kruskal–Wallis H tests were used to compare the diagnostic delay classified by outpatients’ demographic variables. Multivariable linear regression analysis was performed to determine potential factors related to diagnostic delay. Statistical significance was set at *p* < 0.05.

## Results

### Demographic characteristics

**Figure 1:**
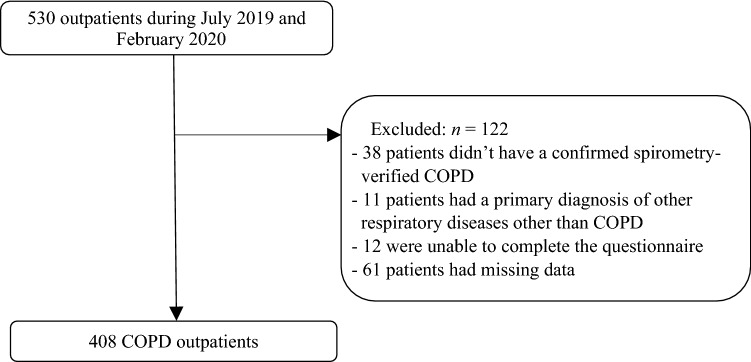
The flowchart of eligible patients. *COPD* chronic obstructive pulmonary disease.

From the original queue including 530 outpatients, 122 patients were excluded. Of these, 38 patients didn’t have a confirmed spirometry-verified COPD, 11 patients had a primary diagnosis of other respiratory diseases other than COPD, 12 were unable to complete the questionnaire, and 61 patients had missing data. Finally, a total of 408 outpatients meeting the eligible criteria were included (Fig. 1).

Table 1 depicts the outpatients’ demographic characteristics. Males constituted 90.2% of the respondent group, while 9.8% were females. Of the included patients, 41.7% had no acute exacerbation, 27.2% had an acute exacerbation, and 31.1% had two or more acute exacerbations in the previous year. Nearly two-thirds of the participants lived in rural areas and 33.1% lived in cities. A vast majority (92.2%) of the patients did not live alone, while 7.8% lived alone. Almost all patients (97.8%) had medical insurance in this study, except for 2.2% who did not. The median duration of diagnostic delay was 230 (50–720) days. Of the respondents, 16.4% chose first-level hospitals for their first visit, 29.9% chose secondary hospitals, and 53.7% chose tertiary hospitals for their first visit. Almost half (47.3%) of the patients underwent pulmonary function tests during their first visit, while 52.7% did not. Moreover, with the increase in hospital level, the proportion of patients undergoing pulmonary function test during the first visit gradually increased (*p* < 0.001) (Table 2).

**Table 1:** Descriptive statistics of the outpatients’ demographic characteristics.

Characteristics	n = 408
**Sex**	
Male	368 (90.2%)
Female	40 (9.8%)
**Age (years)**	65 (58–69)
**Number of acute exacerbations in the previous year**	
0	170 (41.7%)
1	111 (27.2%)
≥ 2	127 (31.1%)
**FEV1% predicted**	53.3 (35.0–69.2)
**CAT score**	13 (8–18)
**mMRC scale**	2 (1–3)
**Residence**	
City	135 (33.1%)
Rural area	273 (66.9%)
**Resident manner**	
Living alone	32 (7.8%)
Not living alone	376 (92.2%)
**Years of education**	9 (6–9)
**Medical insurance**	
Yes	399 (97.8%)
No	9 (2.2%)
**Diagnostic delay (days)**	230 (50–720)
**Hospital classification for the first seeking-treatment visit**	
First-level hospital (I)	67 (16.4%)
Secondary hospital (II)	122 (29.9%)
Tertiary hospital (III)	219 (53.7%)
**Receiving PFT during the first seeking-treatment visit**	
Yes	193 (47.3%)
No	215 (52.7%)

*FEV1* forced expiratory volume in one second, *CAT* COPD Assessment Test, *mMRC*, modified Medical British Research Council, *PFT* pulmonary function test.

**Table 2:** Proportion of patients receiving PFT during the first seeking-treatment visit among different levels of hospitals.

PFT, n (%)	First-level hospital (I) n = 67	Secondary hospital (II) n = 122	Tertiary hospital (III) n = 219	p-value
Yes	1 (1.5%)	30 (24.6%)	162 (74.0%)	< 0.001
No	66 (98.5%)	92 (75.4%)	57 (26.0%)	

*PFT* pulmonary function test.

### Analysis of potential influencing factors associated with diagnostic delay

Tables 3 and 4 report the findings of the Wilcoxon test and the Kruskal–Wallis H analysis, which were performed to explore the differences of diagnostic delay among different characteristics. In terms of diagnostic delay, there were significant differences between rural areas and cities, living alone and not living alone, CAT < 10 and CAT ≥ 10, and mMRC < 2 and mMRC ≥ 2 (all *p* < 0.01). There were also significant differences among participants classified by age, FEV1% predicted, and years of education (all *p* < 0.001).

**Table 3:** Wilcoxon test for demographic differences in diagnostic delay.

Characteristics	Diagnostic delay (days)	p-value
**Sex**		
Male	210 (42–720)	0.209
Female	430 (91–1028)	
**Residence**		
Rural area	300 (90–915)	< 0.001
City	100 (18–390)	
**Resident manner**		
Living alone	660 (248–2645)	0.001
Not living alone	200 (40–680)	
**CAT score**		
< 10	119 (30–353)	< 0.001
≥ 10	300 (90–1080)	
**mMRC scale**		
< 2	95 (28–300)	< 0.001
≥ 2	300 (90–1045)	
**Medical insurance**		
Yes	50 (210–720)	0.102
No	600 (269–1710)	

*CAT*, COPD Assessment Test, *mMRC*, modified Medical British Research Council, *PFT* pulmonary function test.

**Table 4:** Kruskal–Wallis H analysis for demographic differences in diagnostic delay.

Characteristics	Diagnostic delay (days)	p-value
**Age**		
< 50 years	29 (10–125)	< 0.001
50–60 years	200 (50–630)	
60–70 years	210 (38–848)	
≥ 70 years	300 (99–1025)	
**FEV1% predicted**		
< 30%	535 (163–2700)	< 0.001
30—50%	330 (120–1440)	
50—80%	140 (30–540)	
≥ 80%	60 (14–300)	
**Number of acute exacerbations in the previous year**		0.376
0	200 (47–613)	
1	240 (40–930)	
≥ 2	270 (63–900)	
**Years of education**		
≤ 6 years	360 (110–1410)	< 0.001
7 to ≤ 9 years	210 (30–588)	
> 9 years	99 (14–300)	

*FEV1* forced expiratory volume in one second.

Table 5 demonstrates linear regression analysis of potential influencing factors that may be associated with diagnostic delay. Linear regression analysis showed that significant predictors of diagnostic delay included FEV1% predicted (*p* < 0.05), resident manner (*p* < 0.001), and years of education (*p* < 0.01).

**Table 5:** Regression analysis of potential factors related to diagnostic delay.

Variables	Unstandardized coefficients		Standardized coefficients	t	p-value
	B	Standard error			
Sex	− 163.5	301.1	− 0.026	− 0.543	0.587
Age	15.9	11.5	0.067	1.383	0.167
Number of acute exacerbations in the previous year	− 13.5	25.4	− 0.025	− 0.529	0.597
FEV1% predicted	− 10.8	4.9	− 0.130	− 2.216	0.027
CAT score	− 1.7	18.4	− 0.006	− 0.092	0.927
mMRC scale	173.8	125.8	0.094	1.381	0.168
Residence	183.1	201.8	0.045	0.907	0.365
Resident manner	1433.2	340.0	0.203	4.215	< 0.001
Years of education	− 93.4	27.3	− 0.171	− 3.420	0.001
Medical insurance	617.5	616.4	0.048	1.002	0.317

*FEV1* forced expiratory volume in one second, *CAT* COPD Assessment Test, *mMRC* modified Medical British Research Council.

## Discussion

To the best of our knowledge, this is the first study to investigate the diagnostic delay and its influencing factors in COPD patients. Our study indicated that COPD patients had varying degrees of diagnostic delay (median [IQR]: 230 [50–720] days). Another study showed the median delayed diagnosis interval in patients with alpha-1 antitrypsin deficiency was 2.9 years^16^. In a recent retrospective study, it was found that nearly half of the liver cirrhosis patients delayed the diagnosis of hepatocellular carcinoma (HCC) in 60 days or more after danger signs^17^. A new study from the United Kingdom has shown that diagnostic delay may exist in patients with inflammatory bowel disease (IBD); precisely, 92% of the patients were diagnosed within 2 years of the development of the symptoms and 50% were diagnosed within 4 months^18^. The diagnostic delay in COPD patients was relatively long, which might result from COPD being a chronic disease with a longer disease process and gradual aggravation of symptoms.

Previous studies have shown that the proportion of COPD patients who have undergone pulmonary function tests is not high^10,19^. However, these studies did not analyse the relationship between the proportion of patients receiving pulmonary function tests and hospital classifications. As expected, the proportion of patients whose first diagnosis institution was a high-level hospital, who underwent pulmonary function tests, was much higher than that of patients who visited primary hospitals. Therefore, the classification of hospitals is more conducive to understanding the true status of patients’ lung function. In addition, this also illustrates the importance of propagating and popularising lung function tests in primary hospitals.

Our study indicated that the diagnostic delay of outpatients living in rural areas was significantly longer than that for those living in cities, and this may result from that COPD patients living in rural areas have poorer access to medical resource. Our study also found that resident manner was a significantly influencing factor of diagnostic delay in COPD patients. Elderly people living alone have an increased risk of depressive symptoms directly or indirectly by their reduced sense of belonging^20^. We speculated that patients living alone might be due to the lack of supervision from family members or financial constraints, and emotional depression increased the possibility of delay in seeking medical treatments. Interestingly, we found that the higher the disease severity (CAT, mMRC, and FEV1% predicted) of COPD patients, the longer their visits were delayed, which was different from our original assumptions. We thought that symptoms of patients in the early disease stage were mild and had no significant impact on daily life, so patients did not seek medical treatments in time; thus, the diagnostic delay naturally increased as the disease developed. Tejwani et al. also concluded that for each additional year of diagnostic delay, the subject’s FEV1% predicted decreased by 0.3% in patients with alpha-1 antitrypsin deficiency^16^. Besides, our results showed that the diagnostic delay of COPD patients continued to decrease with the increase of education years. A Chinese study reflected that there might be correlation between educational levels and COPD disease risks^21^. We speculated that patients with lower educational level might have poorer awareness of seeking medical care.

This study has some limitations. Firstly, since this study was restricted to outpatients from a single center, the results should be generalised with caution. Future studies should examine other public and private hospitals to more broadly explore diagnostic delay and its influencing factors in patients with COPD. Secondly, the relationship between knowledge level and diagnostic delay was not investigated as there might exist significant variances between newly diagnosed patients and previously diagnosed patients. Thirdly, we didn’t perform interventions targeting influencing factors of diagnostic delay, and future prospective studies are needed to address this issue.

## Conclusions

Our study indicates that varying degrees of diagnostic delay may exist in patients with COPD. Measures are needed to intervene in the potential factors associated with diagnostic delay.
